# Developing a predictive model for neoadjuvant therapy in HER2 overexpression breast cancer using multi-parameter MRI radiomics: two-center retrospective study

**DOI:** 10.3389/fonc.2025.1544058

**Published:** 2025-07-15

**Authors:** Lingling Wang, Jingru Yang, Li Yang, Yun Zhu, Xiaomin Tang, Xinyu Cao, Wenbo Kang, Haitao Sun, Zongyu Xie

**Affiliations:** ^1^ School of Medical Imaging, Bengbu Medical University, Bengbu, China; ^2^ Department of Radiology, The First Affiliated Hospital of Bengbu Medical University, Bengbu, Anhui, China; ^3^ Department of Radiology, Zhongshan Hospital, Shanghai Institute of Medical Imaging, Fudan University, Shanghai, China; ^4^ State Key Laboratory of Bioelectronics, School of Biological Science and Medical Engineering, Southeast University, Nanjing, China

**Keywords:** breast cancer, HER2 overexpression, radiomics, nomograms, magnetic resonance imaging

## Abstract

**AIM:**

To explore an MRI-based radiomics model for predicting the efficacy of neoadjuvant therapy (NAT) for breast cancer with HER2 overexpression.

**Materials and Methods:**

A total of 133 patients with HER2 positive breast cancer who underwent neoadjuvant therapy were retrospectively enrolled and divided into pathological complete response (PCR) and non-PCR groups. The patients from two centers were split into a training group (n=68) and a test group (n=65). MRI sequences (fs-T2WI, DWI, DCE-MRI) were used to outline regions of interest (ROI). Optimal features were selected using f-classif function and LASSO regression, and a multi-parameter MRI radiomics score (Rad-score) was constructed via logistic regression. Clinical independent predictors were identified to build a clinical model, and a nomogram was developed by combining Rad-score with these predictors. Model performance was evaluated using AUC, DeLong test, calibration curves, and decision curve analysis (DCA).

**Results:**

In this study, multivariate analysis identified key predictive clinical factors for pCR, including Ki-67 increment index and tumor morphology. Additionally, a total of 3375 radiomics features were extracted, and 7 key features were selected for model construction. Compared with the image group model and clinical model, the nomogram model based on imaging group had the best predictive performance (training group AUC: 0.894, sensitivity 83.72%, specificity 84.00%, test group AUC: 0.878, sensitivity 88.64%, specificity 71.43%). The calibration and decision curve analyses confirmed its strong consistency and clinical utility compared to individual models.

**Conclusion:**

The nomogram model based on multi-parameter MRI has a steady performance in predicting the efficacy of NAT in breast cancer patients with HER2 overexpression, which provides important guidance for clinical treatment and decision-making.

## Introduction

Breast cancer has become the leading malignancy threatening the health of women in China, with approximately 420,000 new cases and 120,000 deaths annually (1). Among its subtypes, human epidermal growth factor receptor 2 (HER2)-overexpressing breast cancer is particularly aggressive. Initially identified in rat neural tumors, HER2 was later found by Slamon et al. to be amplified or overexpressed in 20%–30% of breast cancers (2). This subtype is characterized by high invasiveness, heterogeneity, and an elevated risk of recurrence and metastasis, often leading to poor prognosis (3, 4).

Neoadjuvant therapy (NAT) is the preferred treatment for HER2 overexpressing breast cancer, resulting in significantly higher rates of pathologic complete remission (pCR), along with extended disease-free survival (DFS) and overall survival (OS), leading to improved prognosis (5–7). While pathological evaluation is the gold standard for assessing NAT response, it depends on post-surgical specimens and is non-repeatable. Imaging, by contrast, offers a non-invasive and comprehensive way to evaluate treatment outcomes early. Magnetic resonance imaging (MRI) is widely utilized to assess breast cancer response to NAT and residual tumor due to its multi-parametric capabilities and absence of ionizing radiation (8, 9). However, previous studies have largely focused on general breast cancer imaging, often neglecting the specific characteristics of HER2 overexpressing breast cancer, a highly heterogeneous subtype with distinct treatment responses and imaging manifestations after NAT. Accurate prediction and evaluation in this group are vital for developing precise treatment plans.

Radiomics, a novel research method, transforms medical images into high-throughput quantitative data, revealing deeper features that are not visible to the naked eye and enabling comprehensive analysis of lesions. This approach increasingly supports personalized cancer treatment by providing detailed insights into quantitative image morphology and spatial distribution (10). The integration of radiomics with pre-treatment MRI has demonstrated potential for accurately predicting neoadjuvant therapy efficacy for breast cancer, paving the way for more precise evaluations and individualized treatment strategies (11, 12). However, research on predicting NAT outcomes for HER2-overexpressing breast cancer using multi-parametric MRI is scarce.

In this study, multi-parametric MRI features, combined with clinical characteristics, were used to develop a prediction model to evaluate NAT outcome prediction performance for HER2-overexpressing breast cancer.

## Materials and methods

This retrospective study was approved by the ethics committee of Bengbu Medical University (No. 372 [2024]); and did not require written informed consent. Clinical trial number: not applicable.

### General information

**Figure 1:**
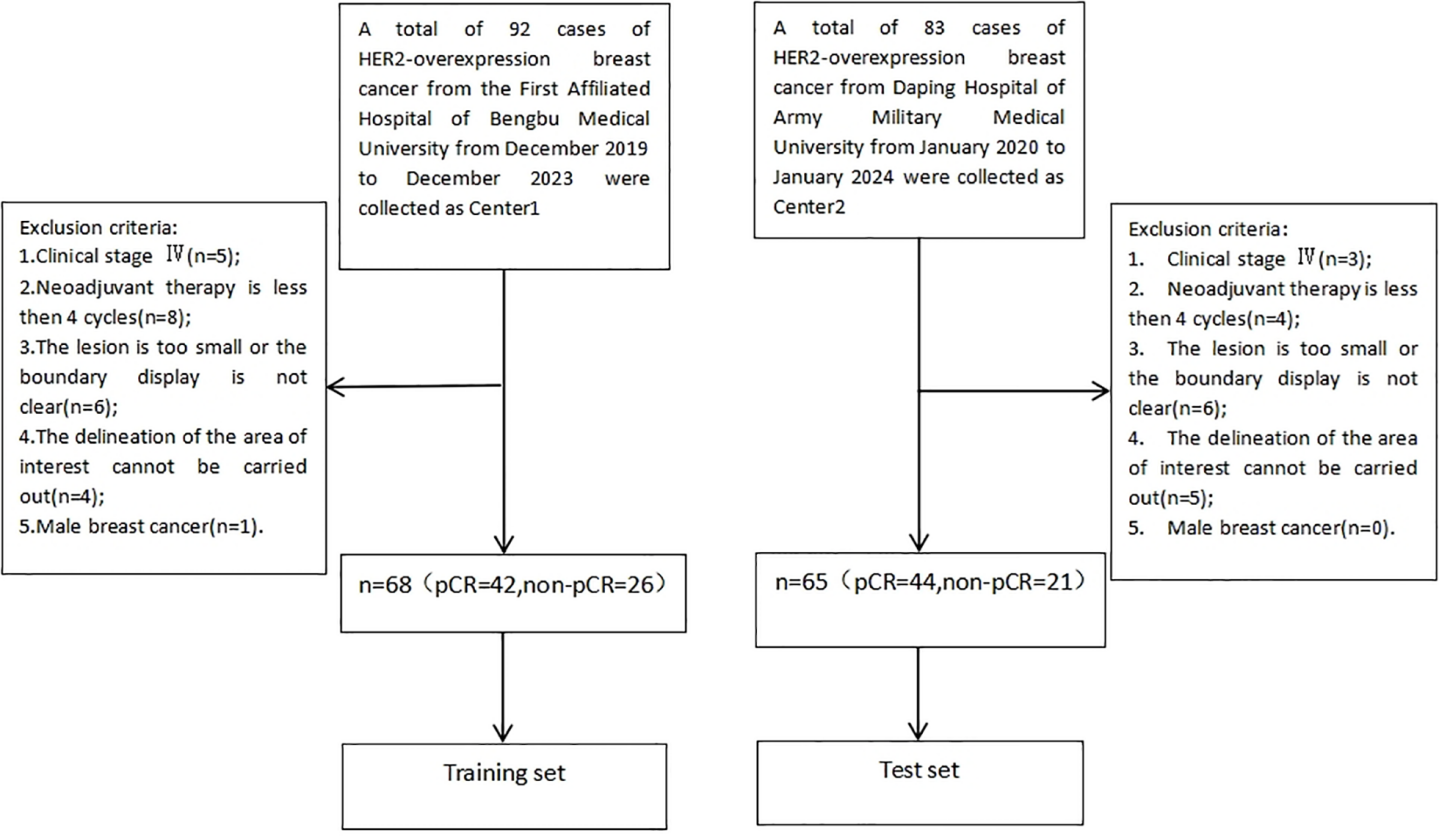
Flow diagram of participants enrollment.

Records of patients with HER2 overexpressing breast cancer who received neoadjuvant therapy between December 2019 and January 2024 at the First Affiliated Hospital of Bengbu Medical University and Daping Hospital of Army Medical University were retrospectively reviewed. The study included patients who were newly diagnosed of HER2 overexpressing breast cancer confirmed by core needle biopsy, received neoadjuvant therapy combined with anti-HER2 therapy, and completed surgical treatment along with postoperative pathological evaluation at either the First Affiliated Hospital of Bengbu Medical University or Daping Hospital of Army Medical University. Patients were excluded if they had clinical stage IV disease, received fewer than 4 cycles of neoadjuvant therapy, had lesions that were too small or poorly defined to delineate the region of interest, or had male breast cancer. Explanation of the exclusion criteria for “ROI delineation cannot be performed” in **Figure 1**:(1) Image quality issues: Severe artifacts in the original images (e.g., susceptibility artifacts caused by metal implants, motion artifacts), low signal-to-noise ratio (e.g., due to improper scanning parameters or poor patient cooperation), or insufficient contrast (e.g., failed contrast-enhanced scans) made it impossible to distinguish the ROI from surrounding tissues. (2) Abnormal or missing anatomical structures: The target organ could not be delineated using conventional imaging methods due to congenital malformations, severe pathological changes (e.g., tumor infiltration leading to the disappearance of organ contours), or post-surgical alterations (e.g., partial/complete organ resection). (3) Technical limitations: The current scanning sequence or imaging plane did not cover the target region (e.g., missing critical slices), or the minimum identifiable size of the ROI could not meet the analysis requirements due to equipment performance constraints (e.g., insufficient spatial resolution).

In total, 133 patients met the criteria, with 68 from the First Affiliated Hospital of Bengbu Medical University designated as the training group and 65 from Daping Hospital of Army Medical University as the test group, further categorized into the PCR group (86 cases) and non-PCR group (47 cases) based on the achievement of pathologic complete remission (PCR) after neoadjuvant therapy (**Figure 1**). PCR was defined as the absence of invasive carcinoma and carcinoma *in situ* in the primary breast, with no residual metastasis in regional lymph nodes (13).

### Examination method

All patients in the study underwent imaging with 3.0T MRI scanners (Philips Achieva, Center 1) and 1.5T MRI scanners (Siemens Magnetom Aera, Center 2). They were positioned prone, with both breasts naturally suspended to align with the center of the breast coil. Patients were instructed to hold their breath and maintain stillness to minimize motion artifacts. Imaging sequences included T1-weighted imaging (T1WI), fat saturation T2-weighted imaging (fs-T2WI), diffusion-weighted imaging (DWI), pre-enhanced T1WI, and dynamic contrast-enhanced (DCE) MRI.

A T1WI plain scan was conducted before contrast agent injection to create a baseline mask image. For the DCE sequence, Gd-DTPA contrast agent was used at a dose of 0.2 mmol/kg in Center 1 and 0.1 mmol/kg in Center 2, injected into the median cubital vein using a high-pressure syringe, followed by 20 mL of normal saline. Scanning continued for each phase, with a duration of 60 seconds per phase. Center 1 collected a total of 5 phases, while Center 2 collected 6 phases. MRI scanning parameters are detailed in **Supplementary Materials Tables S1, S2**.

### Clinical data collection and analysis

Clinical data were collected, including age, menstrual status, neoadjuvant therapy, lymph node enlargement, Ki-67 increment index(Baseline value before treatment), clinical stage of tumor before treatment, and whether pCR was achieved after treatment (**Table 1**). The clinical staging is based on the American Joint Committee Cancer (AJCC) Breast Cancer Staging System (8th Edition) (14). The new adjuvant treatment plan refers to the National Comprehensive Cancer Network Guide 2020 edition (15). The positive criteria of HER2 were immunohistochemical HER2 (3+) or HER2 (2+) and fluorescence *in situ* hybridization (FISH) (16). All cases involved in the study were treated with standard NAT regimens and treatment cycles to minimize their impact on the results of the study. NAT regimens follow NCCN treatment guidelines for breast cancer (17).

**Table 1:** Clinical and radiological baseline characteristics of HER2-overexpression breast cancer in the training and test cohorts.

Characteristics	Training set (n=68)				Test set (n=65)			
	None-pCR (n=26)	pCR(n=42)	Statistical value	*P*-Value	None-pCR (n=21)	pCR(n=44)	Statistical value	*P*-Value
Age	50.44 ± 8.43	53.38 ± 9.74	0.942	0.214	50.38 ± 8.71	52.59 ± 9.60	0.024	0.375
Menopausal state			0.930	0.335			0.253	0.615
No	13 (50.0)	16 (38.1)			9 (42.9)	16 (36.4)		
Yes	13 (50.0)	26 (61.9)			12 (57.1)	28 (63.6)		
lymphadenopathy			0.169	0.681			0.018	0.894
No	4 (15.4)	5 (11.9)			17 (81.0)	35 ( 79.5)		
Yes	22 (84.6)	37 (88.1)			4 (19.0)	9 (20.5)		
MRI maximum tumor diameter	38.62 ± 15.86	38.60 ± 15.46	-0.069	0.945	31.0 (14.0, 89.0)	27.5 (9.0, 77.0)	-1.278	0.201
Tumor margin			0.269	0.604			3.155	0.076
Clear	7 (26.9)	9 (21.4)			0 (0.0)	6 (13.6)		
Unclear	19 (7301)	33 (78.6)			21 (100.0)	38 (86.4)		
Tumor morphology			6.477	0.011			1.646	0.034
regular	3 (11.5)	17 (40.5)			2 (9.5)	10 (22.7)		
irregular	23 (88.5)	25 (59.5)			19 (90.5)	34 (77.3)		
Enhancement patterns			2.352	0.125			0.032	0.857
Lump-like enhancement	20 (76.9)	38 (90.5)			10 (47.6)	22 (50.0)		
Non-lump-like enhancement	6 (23.1)	4 (9.5)			11 (52.4)	22 (50)		
TIC curve			0.931	0.628			2.039	0.361
I	4 (15.4)	5 (11.9)			0 (0.0)	4 (9.1)		
II	9 (34.6)	11 (26.2)			7 (33.3)	13 (29.5)		
III	13 (50.0)	26 (61.9)			14 (66.7)	27 (61.4)		
ADC value	0.9 (0.6, 1.2)	1.0 (0.1, 1.5)	0.419	0.040	0.8 (0.4, 1.7)	1.0 (0.4, 1.5)	-2.302	0.021
Number of lesions			2.875	0.090			0.147	0.702
Single	17 (65.4)	35 (83.3)			19 (90.5)	41 (93.2)		
Multiple	9 (34.6)	7 (16.7)			2 (9.5)	3 (6.8)		
Ki-67 express			8.594	0.003			8.313	0.004
No	13 (50.0)	7 (16.7)			13 (61.9)	11 (25.0)		
Yes	13 (50.0)	35 (83.3)			8 (38.1)	33 (75)		
Clinical T stage			1.513	0.679			2.604	0.457
T1	0 (0.0)	2 (4.8)			1 (4.8)	1 (2.3)		
T2	19 (73.1)	28 (66.7)			13 (61.9)	35 (79.5)		
T3	6 (23.1)	11 (26.2)			6 (28.6)	6 (13.6)		
T4	1 (3.8)	1 (2.4)			1 (4.8)	2 (4.5)		
BPE			4.484	0.214			8.690	0.034
Seldom	1 (3.8)	9 (21.4)			19 (90.5)	35 (79.5)		
Mild	14 (53.8)	21 (50.0)			0 (0.0)	7 (15.9)		
Moderate	9 (34.6)	9 (21.4)			2 (9.5)	0 (0.0)		
Severe	2 (7.7)	3 (7.1)			0 (0.0)	2 (4.5)		

TIC, time-signal curve; BPE, background parenchymal enhancement. *P* < 0.05 is considered statistically significant.

### MR image analysis

MR images were evaluated by two radiologists with over 5 years of experience in breast MRI diagnosis using a post-processing workstation. The maximum diameter of the lesions was measured on the T1 image 90 seconds after injection of contrast medium, and the enhancement mode of the lesions, whether the axillary lymph nodes and skin were invaded, whether the edge of the lesions was clear, whether the shape was regular, the number of lesions, the apparent diffusion coefficient (ADC), the background parenchymal enhancement (BPE) and the type of time-signal intensity curve (TIC) were recorded. BPE is defined as enhancement of normal fibrogland in breast. According to BI-RADS, TIC can be divided into three types: inflow type-that is, slow and continuous enhancement; A small number will show a downward trend in the late stage; Platform type-dynamic signal intensity reaches its peak in early stage, and has no obvious change in delay period; Outflow type-dynamic early signal intensity reaches its peak and then decreases. Discrepancies in interpretation were resolved through consensus discussion.

**Figure 2:**
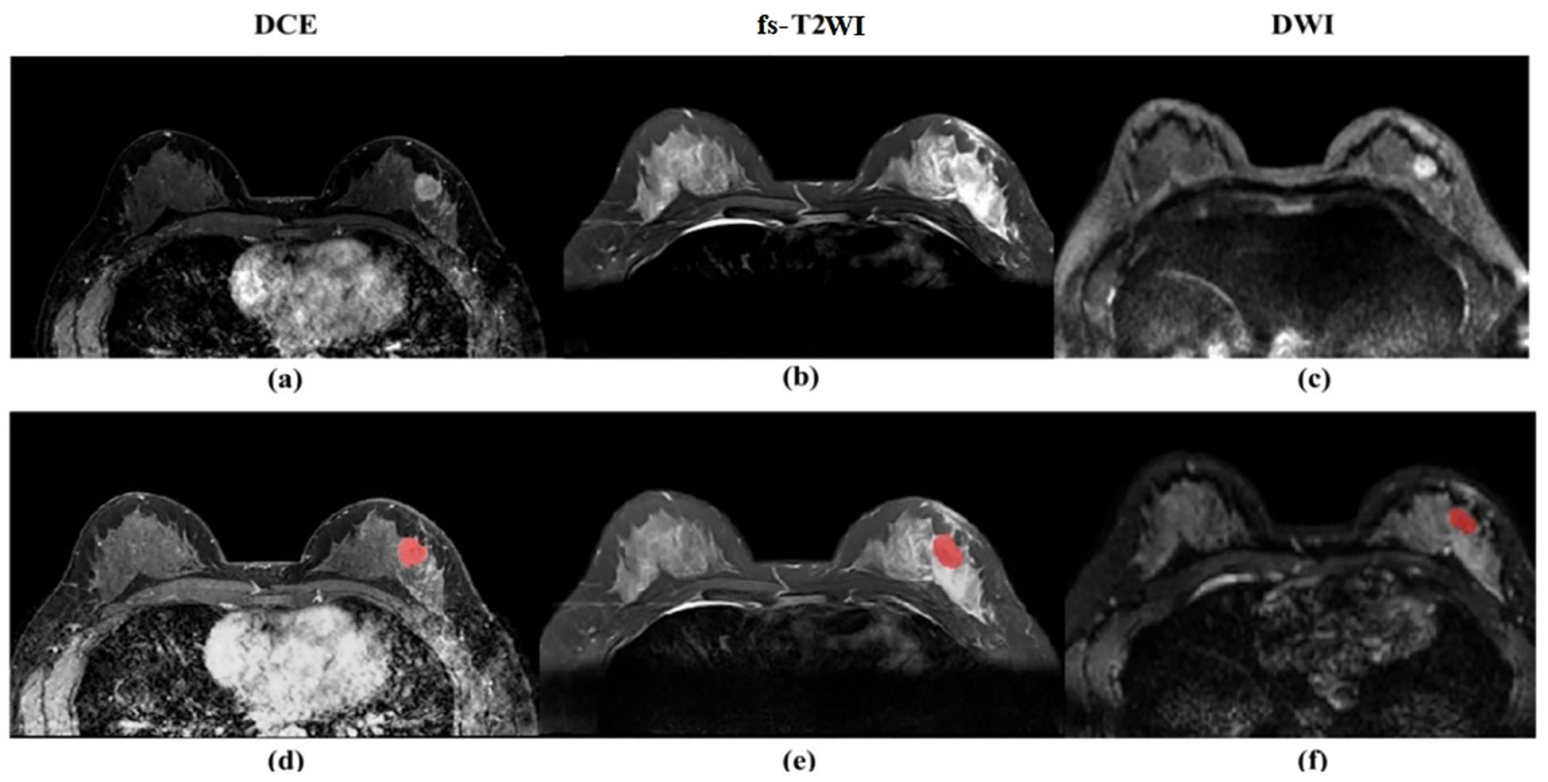
**(a)** Dynamic scanning is performed after intravenous injection of contrast agent to evaluate the blood supply and vascular permeability of the lesion. The enhanced area (white high signal) indicates vascular richness or abnormal proliferation. **(b)** High signal (white) indicates the lesion area. **(c)** Detects the degree of restricted diffusion of water molecules, with high signal (white) indicating the lesion area. **(d)** Dynamic scanning is performed after intravenous injection of contrast agent to evaluate the blood supply and vascular permeability of the lesion. The enhanced area (white high signal) indicates vascular richness or abnormal proliferation. **(e)** High signal (white) indicates the lesion area. **(f)** detects the degree of restricted diffusion of water molecules, with high signal (white) indicating the lesion area, and red markers indicating the manually annotated lesion area of interest.

The second-phase images of fs-T2WI, DWI and DCE-MRI sequences from enrolled patients were imported into Darwin Intelligent Research Platform (http://premium.darwin.yizhun-ai.com) in DICOM format, and an imaging doctor manually delineated the largest layer of tumor, and then delineated the region of interest (ROI) of the second phase images of DCE-MRI layer by layer to form 3D-ROI (**Figure 2**). The reason for choosing this stage to delineate layer by layer is that the enhancement of the lesion reaches the maximum currently, and the background parenchyma enhancement of normal glands is relatively light, and the contrast between the lesion and surrounding glands is enhanced, which is more conducive to the display of the lesion. In cases of disagreement, a third radiologist evaluated the delineations, and consensus was reached through discussion.

### Feature extraction and screening

After image segmentation, features were extracted and processed, and finally 1125 original radiomics features were extracted from fs-T2WI, DWI and DCE-MRI sequences, totaling 3375. The features mainly include first-order features, texture features, and higher-order statistical features. The extracted feature values are preprocessed to [0, 1] by Maximum and Minimum Normalization, then intra-class correlation coefficient (ICC) is calculated, and the features with ICC > 0.75 are retained, that is, the features with good consistency. Using the least absolute shrinkage and selection operator (LASSO) regression, 10-fold cross-validation was performed on the training set to select the most predictive radiomics features.

### Construction of radiomics model

A multi-parameter MRI model (composed of DCE-2 features, fs-T2WI features and DWI features) was constructed using logistic regression (LR) based on the imaging features selected from the second phase images of fs-T2WI sequences, DWI sequences and DCE-MRI sequences. The diagnostic efficacy of the model was evaluated by the area under curve (AUC) of the subject working characteristics (ROC), and the results were converted into radiomics score (Rad-score). The whole process is firstly carried out in the training group data set, and then the external verification of data is completed in the test group data set.

### Building federation model

**Figure 3:**
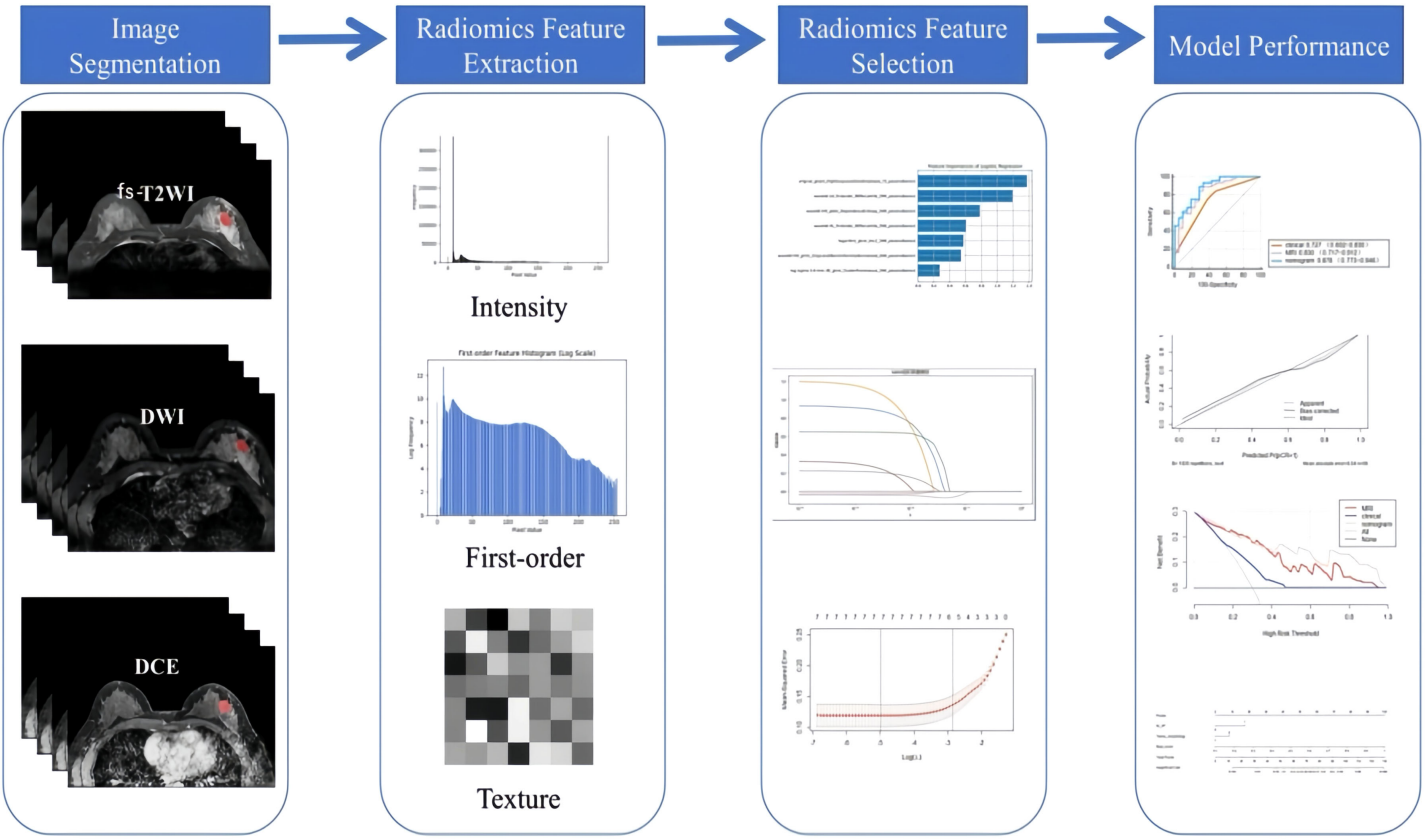
An overview of MRI-based radiomics analysis.

Through uni-/multi-variate logistic regression analysis, the independent risk factors predicting the efficacy of neoadjuvant therapy for breast cancer with HER2 overexpression were screened, and the clinical model was established. Based on the combination of clinical independent risk factors and multi-parameter MRI radiomics model, the nomogram model was established to visualize the data. The workflow is shown in **Figure 3**. (Note: In the figure, “Intensity features” and “First-order features” describe the same type of features, with different names adopted due to differing expression conventions in application scenarios).

### pathological evaluation

According to Miller-Payne (MP) histological grading system, the surgical specimens of patients who completed neoadjuvant therapy were evaluated pathologically. G1-G4 were classified as non-pathological complete remission (non-pCR) group, and G5 as pathological complete remission (pCR) group. The evaluation criteria (18)are as follows:

G1: There was no decrease in tumor cells;
G2: The number of tumor cells decreased slightly, the reduction ratio was less than or equal to 30%, and more tumor cells were still visible;
G3: The reduction ratio of tumor cells is between 30% and 90%;
G4: Tumor cells decreased significantly, with a reduction ratio of ≥ 90%, leaving only small clusters or scattered single tumor cells;
G5: No invasive cancer cells with or without residual ductal carcinoma *in situ* (DCIS) were found on all pathological sections.

### Statistical analysis

Statistical analyses were performed in SPSS 27.0, MedCalc 19.1.3, and *R* software (Version 4.2.3). As this was a retrospective study aimed at exploring the potential predictive value of radiomic features rather than validating clinical endpoints, no priori sample size calculation was performed. The measurement data conforming to the normal distribution were expressed as mean ± standard deviation (x ± s) and were compared between groups using the T-test of two independent samples. Measurement data inconsistent with normal distribution are presented as median (upper quartile, lower quartile) and analyzed by Mann⁃Whitney U test between groups. Counting data are expressed in frequency and compared using chi-square test. The ROC curve was drawn by Medcalc 19.1.3 software, and the AUC, accuracy, sensitivity and specificity of the model were calculated. *R* software (Version 4.2.3) was used to construct alignment chart, calibration curve (CC) and decision curve analysis (DCA) to evaluate the goodness of fit and clinical value of different models.

## Results

### Clinical data analysis

This study included 133 patients, divided into a training set of 68 patients and a test set of 65 patients. The training set comprised 42 patients with pathologic complete remission (PCR) and 26 without PCR, while the test set included 44 patients with PCR and 21 without PCR. No significant differences were found in the distribution of clinical and imaging features between the training and test sets (P > 0.05).

### Clinical features and model construction

Using pathological evaluation as the gold standard, candidate variables were screened through univariate analysis in the training set; the selected variables were then incorporated into multivariate logistic regression analysis of the training set to determine the final independent predictors Ki-67 proliferation index and tumor morphological characteristics to construct the model; the established model was directly applied to the test set to calculate prediction probabilities and evaluate performance (**Table 2**).

**Table 2:** Uni- and multi-variate logistic regression analysis of HER2- overexpression breast cancer in training set.

Parameters	Univariate analysis		Multivariate analysis	
	OR value (95%CI)	*p* value	OR value (95%CI)	*p* value
Age(years)	1.035 (0.980-1.093)	0.214		
Menopausal state, premenopausal vs postmenopausal	1.102 (0.856-1.195)	0.893		
Lymphadenopathy, absent vs present	2.647 (0.135-51.806)	0.521		
MRI maximum tumor diameter(mm)	0.906 (0.771-1.065)	0.232		
Tumor margin, clear vs unclear	2.624 (0.130-52.881)	0.529		
Tumor morphology, regular vs irregular	0.039 (0.001-1.588)	**0.046**	0.045 (0.017-0.957)	**0.045**
Enhancement patterns, lump-like enhancement vs non-lump-like enhancement	0.428 (0.026-6.911)	0.550		
TIC curve, I vs II/III	0.366 (0.007-19.988)	0.623		
ADC value	0.033 (0.000-2.673)	**0.028**	16.884 (0.934-305.094)	0.056
Number of lesions, single vs multiple	0.334 (0.021-5.238)	0.435		
Ki-67 express, <20% vs ≥20%	19.904 (1.596-248.176)	**0.020**	5.858 (1.497-22.919)	**0.011**
Clinical T stage, T1 vs T2/T3/T4	0.76 (0.002-10.189)	0.351		
BPE, seldom vs mild/moderate/severe	0.033 (0.001-2.673)	0.128		
Rad-score	17.277 (6.564-45.476)	**0.015**	13.534 (30.705-59.571)	**0.002**

TIC, time-signal curve; BPE, background parenchymal enhancement; Rad-score: radiomics score; OR, odds ratio; CI, confidence interval. *P* < 0.05 is considered statistically significant.

The bold values represent statistically significant values, with a P value less than 0.05.

### Radiomics features and establishment of multi-parameter MRI radiomics model

**Figure 4:**
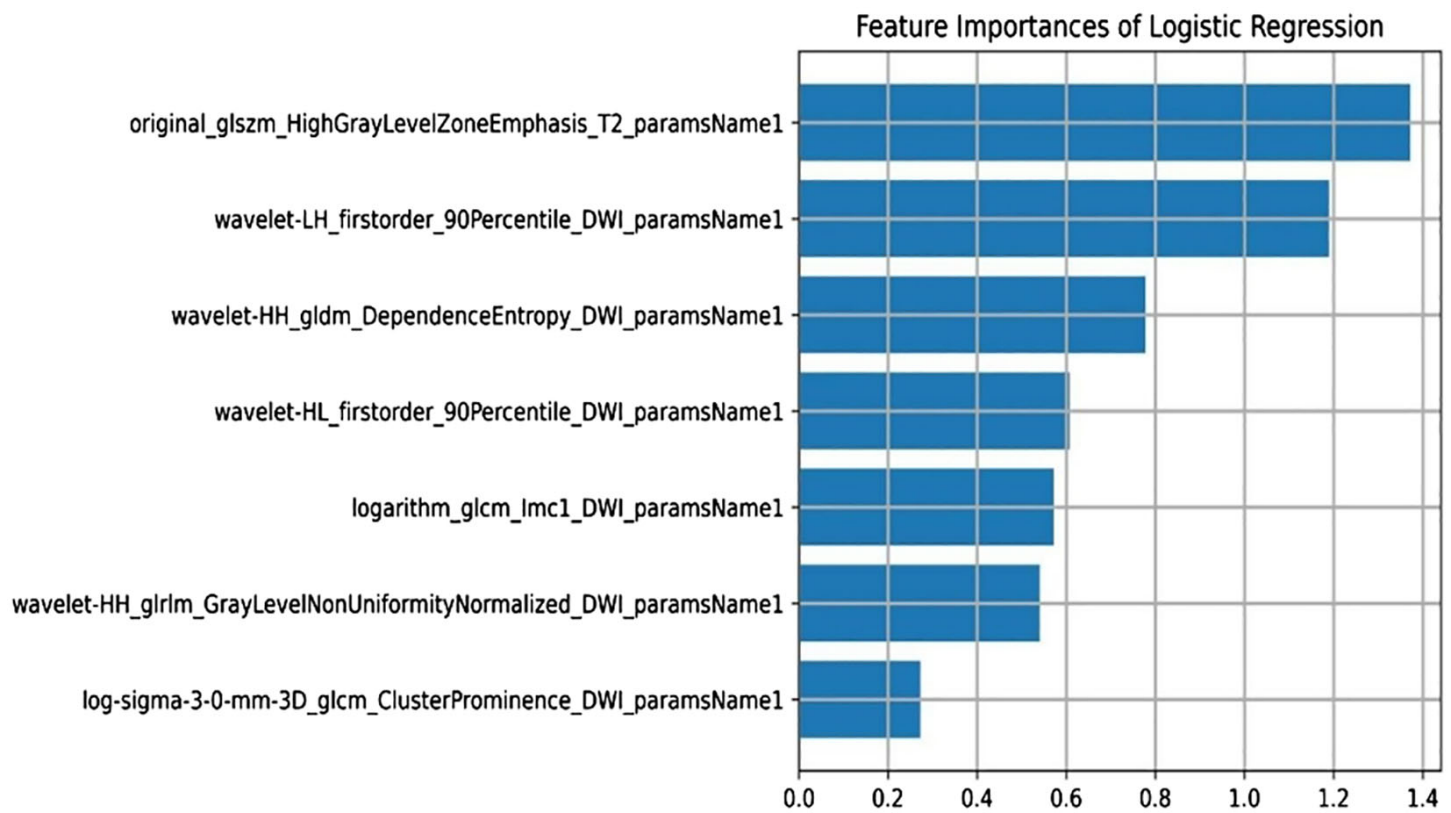
Radiomics features and establishment of multi-parameter MRI radiomics model.

A total of 3375 features were extracted from multi-parameter MRI radiomics. After dimensionality reduction by f-calssif function and LASSO regression, 7 radiomics features were screened out to establish omics model, which was sorted according to weights as shown in **Figure 4**.

Among them, wavelet-LH_first-order_90th percentile_DWI_parameter name 1 and wavelet-HL_first-order_90th percentile_DWI_parameter name 1 are first-order features.WaveletHH_gldm_DependenceEntropy_DWI_paramsName1,logarithm_glcm_Imc1_DWI_paramsName1,wavelet-HH_glrlm_GrayLevelNonUniformityNormalized_DWI_paramsName1 and log-sigma-3-0-mm-3D_glcm_ClusterProminence_DWI_paramsName1 are texture features.Original_glszm_HighGrayLevelZoneEmphasis_T2_paramsName1 are high-order features.

### Establishment of a nomogram model

**Figure 5:**
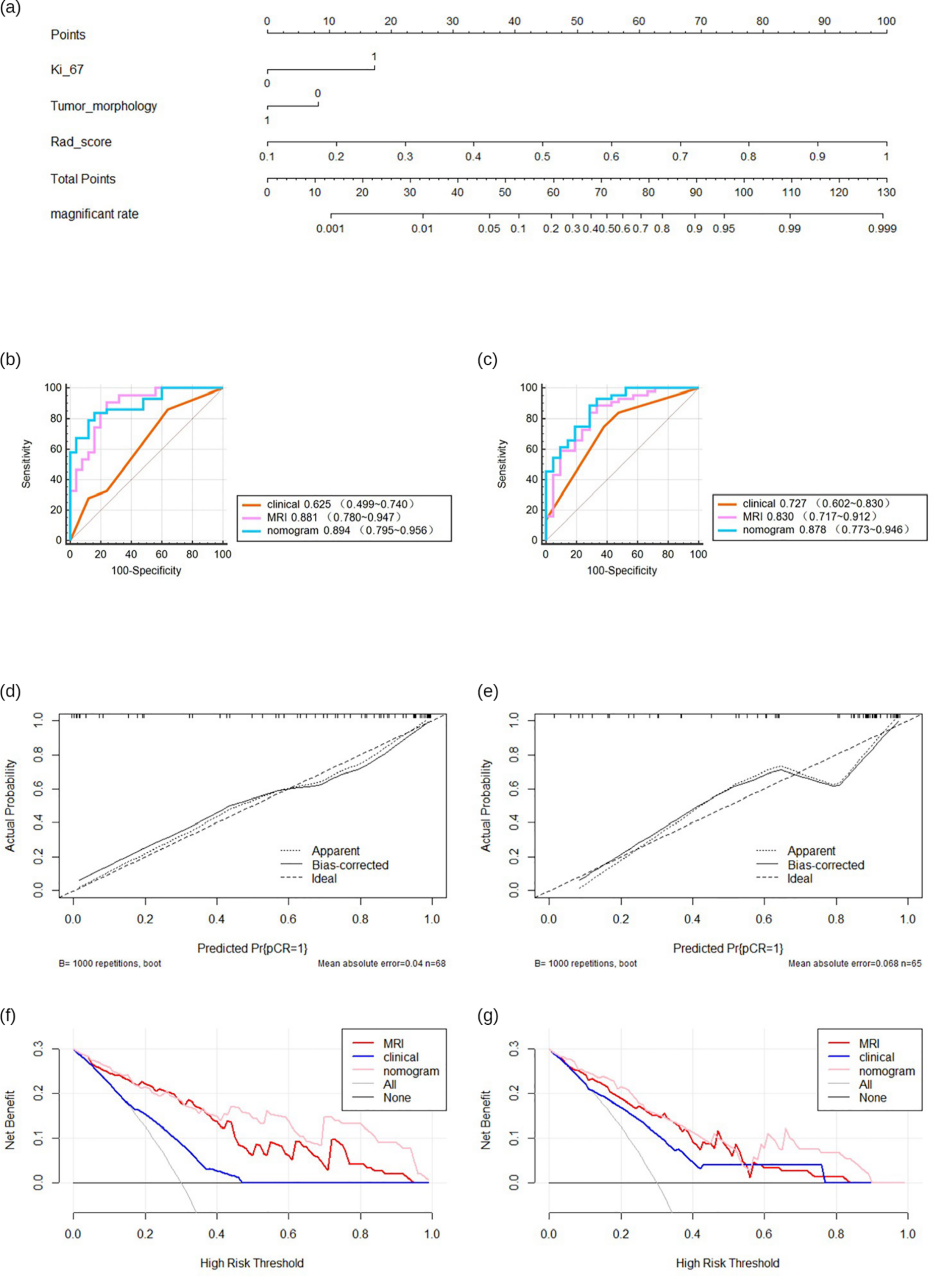
The predictive performance of various models. Nomogram of developed radiomics nomogram **(a)**. Receiver operating characteristics (ROC) curves **(b, c)**, calibration curve **(d, e)**, decision curve **(f, g)** in both training.

Combining the identified clinical independent risk factors with the multi-parameter MRI radiomics model, a nomogram model was developed and visualized (**Figure 5a**). ROC curves were generated to assess the performance of each model in predicting pathological complete remission (pCR) in both the training and test sets. In the training set, the AUCs for the clinical model, multi-parameter MRI radiomics model, and nomogram model were 0.625, 0.881, and 0.894, respectively. In the test set, the AUCs were 0.727, 0.830, and 0.878, respectively (**Table 3**, **Figures 5b, c**). The nomogram model demonstrated the highest predictive performance. The calibration curves indicate that the ideal, predicted, and corrected curves of the nomogram model are closely aligned, reflecting good consistency (**Figures 5d, e**). Additionally, the decision curve analysis shows that the nomogram model outperforms both the multi-parameter MRI radiomics model and the clinical model in terms of patient risk assessment and clinical benefit (**Figures 5f, g**). The DeLong test confirmed that the nomogram exhibits strong consistency across both the training and test cohorts (**Table 4**).

**Table 3:** Performance of three models in training and test cohorts.

Group	Model	AUC	95%CI	Sensitivity	Specificity
Training set	Clinical model	0.625	0.499-0.740	0.860	0.360
	Radiomics MRI model	0.881	0.780-0.947	0.907	0.760
	Radiomics nomogram	0.894	0.795-0.956	0.837	0.840
Test set	Clinical model	0.727	0.602-0.830	0.750	0.619
	Radiomics MRI model	0.830	0.717-0.912	0.841	0.714
	Radiomics nomogram	0.878	0.773-0.946	0.886	0.714

AUC, area under the curve; CI, confidence interval.

**Table 4:** Comparison of ROC curves in the training and test cohorts.

Group	Compares	Z statistic	*p*-Value
Training set	Clinical model versus Radiomics MRI model	3.039	0.002
	Clinical model versus Radiomics nomogram	4.966	<0.001
	Radiomics MRI model versus Radiomics nomogram	0.258	0.797
Test set	Clinical model versus Radiomics MRI model	1.269	0.204
	Clinical model versus Radiomics nomogram	2.904	0.004
	Radiomics MRI model versus Radiomics nomogram	1.245	0.213

*p* is derived from Delong test between each of the ROCs, and *P* < 0.05 is considered statistically significant.

## Discussion

HER2 overexpression breast cancer is characterized by specific therapeutic targets and drugs, making it a focal point of research. Neoadjuvant therapy (NAT) incorporating anti-HER2 targeted therapies has emerged as a cornerstone treatment (19). However, few studies have focused on the relationship between the efficacy of neoadjuvant therapy and the clinical-imaging features or imaging features of HER2 overexpression breast cancer. In this study, we developed a multi-parametric MRI radiomics model to predict the efficacy of NAT in patients with HER2 overexpression breast cancer. The nomogram, integrating the radiomics score (Rad-score) with clinical and imaging features, demonstrated superior performance, with an area under the receiver operating characteristic curve (AUC) of 0.894 (95% CI: 0.796–0.956) in the training cohort and 0.878 (95% CI: 0.773–0.946) in the test cohort. These results indicate that it has the potential to be a non-invasive tool for predicting NAT efficacy in HER2 overexpression breast cancer.

In this study, Ki-67 proliferation index and tumor morphology were significantly correlated with the curative effect of NAT according to clinical and imaging features, which indicated that they have certain potential value in predicting the curative effect of NAT. Ki-67, first identified by Gerdes et al. at Kiel University in Germany while studying nuclear antigens in a Hodgkin’s lymphoma cell line, is a monoclonal antibody marker expressed during mitosis in the cell cycle. It is commonly used to assess tumor cell proliferation and treatment sensitivity (20).The grading criteria for Ki-67 in breast cancer are primarily based on the St. Gallen International Breast Cancer Conference consensus (2013-present) and the WHO classification guidelines for breast cancer. A Ki-67 index of 20% is widely adopted as the cutoff value for proliferative activity, used to distinguish between “low proliferation” and “high proliferation” tumor subtypes. Bae SJ et al. showed that the patients with high expression of Ki-67 in HER2 overexpression breast cancer are more likely to achieve pCR and the prognosis of the patients with high expression is also better (21). Another study showed DFS and OS of patients with high expression level of Ki-67 after NAT were lower than those with low expression level (22). These findings underscore the close relationship between Ki-67 expression and NAT efficacy in HER2-overexpressing breast cancer.

This study also identified a correlation between tumor morphology and the efficacy of NAT, with irregular tumor shapes being associated with a higher likelihood of achieving a pCR. The criteria for “regular morphology” and “irregular morphology” primarily stem from the core features of morphological assessment, wherein tumor morphology is defined based on radiological evaluation by referencing whether the boundary between the tumor and surrounding normal tissue is clear; whether the tumor interior is homogeneous; and whether the tumor exhibits expansive growth (pushing adjacent tissues) or infiltrative growth (invading neighboring structures). The dichotomous classification rule is determined by synthesizing the aforementioned three features: if at least two features are “clear boundary + homogeneous interior + expansive growth,” it is defined as “regular morphology”; if at least two features are “blurred boundary + heterogeneous interior + infiltrative growth,” it is defined as “irregular morphology”; if the feature distribution is ambiguous (e.g., only one feature aligns with “regular” or “irregular”), two radiologists (each with ≥5 years of experience in tumor imaging diagnosis) independently review the case. In instances where the two physicians disagree on the interpretation, a consensus is reached through discussion.

This association may be attributed to the nature of HER2 overexpressing breast cancers, which are characterized by irregularly shaped masses. The amplification of proto-oncogenes in these tumors leads to accelerated cell proliferation, making them more susceptible to the effects of chemotherapy and targeted therapies. This increased sensitivity results in enhanced cell necrosis and apoptosis, thereby facilitating the achievement of pCR following NAT, which is consistent with previous study (23).

There is a pressing need for a non-invasive, reliable method to accurately predict NAT efficacy prior to surgery. Such a method would enable the identification of patients likely to achieve a pathological complete response (pCR), minimize unnecessary treatments, reduce related complications, and improve patient outcomes. However, consensus on the optimal approach for assessing intratumor heterogeneity in clinical practice remains lacking. Furthermore, HER2 expression status determined from core biopsy specimens taken from multiple tumor sites provides only limited insight into the overall heterogeneity of the tumor (24). In contrast, MRI radiomics analysis can evaluate the whole tumor non-invasively and reflect the heterogeneity within the tumor through the spatial distribution of voxel intensity. Some studies have shown that the histological features of MRI images are related to the difference of pathological reactions after NAT (25). For instance, one study integrated multi-parametric MRI to predict pCR in breast cancer patients after NAT, while another developed a radiomics model combining T2WI and DCE sequences, demonstrating strong predictive performance, while another developed a radiomics model combining T2WI and DCE sequences, demonstrating strong predictive performance (11, 26). In this study, the LASSO method identified seven key image features with significant correlation (**Figure 4**), with GLSZM HighGrayLevelZoneEmphasis ranking highest. GLSZM measures the distribution of gray levels and reflects tumor texture heterogeneity—higher values indicate greater variability in tumor texture (26). The second-ranked feature quantifies the distribution of gray values within the tumor, with higher values suggesting more active biological behavior and a higher risk of deterioration. Additionally, the gray level dependence matrix (GLDM), a second-order texture feature derived from wavelet transform, quantifies gray area distribution and also contributes significantly (27). These features, which capture tumor heterogeneity from various dimensions, may be associated with responses to NAT. Since the feature screening process may eliminate all features of a certain sequence in LASSO selection, the highest-ranked feature from its univariate analysis will be forcibly retained to ensure multimodal representation. The primary strength of T1WI lies in its clear visualization of anatomical structures (e.g., tumor boundaries and adjacent organ invasion), making it ideal for morphological evaluation. However, this study focuses on functional radiomics features (e.g., texture) to capture tumor heterogeneity, which is less effectively represented by T1WI signal intensity, as it primarily reflects tissue proton density and longitudinal relaxation time.

Additionally, fs-T2WI emphasizes texture feature extraction (GLCM, GLSZM), reflecting the complexity and heterogeneity of internal tumor structures; DWI primarily extracts diffusion tensor features and higher-order statistics, reflecting cellular density and microstructure; DCE-MRI mainly extracts spatiotemporal joint features, reflecting angiogenic activity. Given that T1WI lacks distinct advantages in these tasks, it was not selected as the primary modality for feature extraction.

In our study, a radiomics model incorporating fs-T2WI, DWI, and DCE-MRI sequences were developed. Fs-T2WI sequences facilitate the detection of abnormal signals associated with cystic necrosis, DWI provides sensitive and accurate measurement of water molecule diffusion in pathological tissues, and DCE-MRI assesses tumor blood flow. The model demonstrated strong predictive performance with an AUC of 0.881 in the training set and 0.830 in the test set. Multivariate regression analysis, integrating clinical independent risk factors with the multi-parameter MRI radiomics model, led to the creation of a nomogram. This nomogram achieved AUCs of 0.894 in the training group and 0.878 in the test group, outperforming the clinical model, which had AUCs of 0.625 and 0.727, respectively. These results underscore the strength of radiomics in revealing multidimensional information not discernible to the naked eye.

There are some limitations in this study. First, this was a retrospective study and there may have been some bias in patient selection. Second, the patient sample size was small, and although the multicenter study was validated by external validation data, more large-scale multicenter prospective studies are needed in the future to verify the predictive performance of the nomogram. Third, the pathological types of HER2 overexpressed breast cancer included in the study were mainly invasive ductal carcinoma, and future studies should include various types of breast cancer to improve the accuracy of the model.

In conclusion, the nomogram model that integrates rad-score with clinical and radiological features demonstrates superior predictive performance. As further prospective studies validate these findings, multi-parametric MRI-based radiomics features are anticipated to offer valuable clinical insights for preoperative assessment and evaluation of NAT efficacy in HER2 overexpressing breast cancer patients. This approach will support clinicians in tailoring individualized, precision treatment plans.

## Data Availability

The original contributions presented in the study are included in the article/**Supplementary Material**. Further inquiries can be directed to the corresponding authors.

## References

[1] Xia C Dong X Li H Cao M Sun D He S. Cancer statistics in China and United States, 2022: profiles, trends, and determinants. Chin Med J (Engl). (2022) 135:584–90. doi: 10.1097/CM9.0000000000002108, PMID: 35143424 PMC8920425

[2] Slamon DJ Clark GM Wong SG Levin WJ Ullrich A McGuire WL. Human breast cancer: correlation of relapse and survival with amplification of the HER-2/neu oncogene. Science. (1987) 235:177–82. doi: 10.1126/science.3798106, PMID: 3798106

[3] Elshazly AM Gewirtz DA. An overview of resistance to Human epidermal growth factor receptor 2 (Her2) targeted therapies in breast cancer. Cancer Drug Resist. (2022) 5:472–86. doi: 10.20517/cdr.2022.09, PMID: 35800378 PMC9255238

[4] Zhang Q Xiu B Wu J. Progress of important clinical research of breast cancer in China in 2023. China Oncol. (2024) 34:135–42. doi: 10.19401/j.cnki.1007-3639.2024.02.001

[5] Burstein HJ Curigliano G Thürlimann B Weber WP Poortmans P Regan MM. Customizing local and systemic therapies for women with early breast cancer: the St. Gallen International Consensus Guidelines for treatment of early breast cancer 2021. Ann Oncol. (2021) 32:1216–35. doi: 10.1016/j.annonc.2021.06.023, PMID: 34242744 PMC9906308

[6] Zhao F Huo X Wang M Liu Z Zhao Y Ren D. Comparing biomarkers for predicting pathological responses to neoadjuvant therapy in HER2-positive breast cancer: A systematic review and meta-analysis. Front Oncol. (2021) 11:731148. doi: 10.3389/fonc.2021.731148, PMID: 34778044 PMC8581664

[7] Orsaria P Grasso A Ippolito E Pantano F Sammarra M Altomare C. Clinical outcomes among major breast cancer subtypes after neoadjuvant chemotherapy: impact on breast cancer recurrence and survival. Anticancer Res. (2021) 41:2697–709. doi: 10.21873/anticanres.15051, PMID: 33952501

[8] Wekking D Porcu M De Silva P Saba L Scartozzi M Solinas C. Breast MRI: clinical indications, recommendations, and future applications in breast cancer diagnosis. Curr Oncol Rep. (2023) 25:257–67. doi: 10.1007/s11912-023-01372-x, PMID: 36749493

[9] Chen S Che S Li J. Progress of MRI and Radiomics in predicting the response to neoadjuvant therapy for breast cancer in different molecular subtypes. Chin J Magn Reson Imaging. (2023) 14:156–60. doi: 10.12015/issn.1674-8034.2023.06.028

[10] Guiot J Vaidyanathan A Deprez L Zerka F Danthine D Frix A. A review in radiomics: Making personalized medicine a reality via routine imaging. Med Res Rev. (2022) 42:426–40. doi: 10.1002/med.21846, PMID: 34309893

[11] Liu Z Li Z Qu J Zhang R Zhou X Li L. Radiomics of multiparametric MRI for pretreatment prediction of pathologic complete response to neoadjuvant chemotherapy in breast cancer: A multicenter study. Clin Cancer Res. (2019) 25:3538–47. doi: 10.1158/1078-0432.CCR-18-3190, PMID: 30842125

[12] Pesapane F Rotili A Botta F Raimondi S Bianchini L Corso F. Radiomics of MRI for the prediction of the pathological response to neoadjuvant chemotherapy in breast cancer patients: A single referral centre analysis. Cancers (Basel). (2021) 13:4271. doi: 10.3390/cancers13174271, PMID: 34503081 PMC8428336

[13] Takada M Toi M. Neoadjuvant treatment for HER2-positive breast cancer. Chin Clin Oncol. (2020) 9:32. doi: 10.21037/cco-20-123, PMID: 32527117

[14] Giuliano AE Connolly JL Edge SB Mittendorf EA Rugo HS Solin LJ. Breast Cancer-Major changes in the American Joint Committee on Cancer eighth edition cancer staging manual. CA Cancer J Clin. (2017) 67:290–303. doi: 10.3322/caac.21393, PMID: 28294295

[15] Kuerer HM Smith BD Krishnamurthy S Yang WT Valero V Shen Y. Eliminating breast surgery for invasive breast cancer in exceptional responders to neoadjuvant systemic therapy: a multicentre, single-arm, phase 2 trial. Lancet Oncol. (2022) 23:1517–24. doi: 10.1016/S1470-2045(22)00613-1, PMID: 36306810

[16] Hassett MJ Li H Burstein HJ Punglia RS. Neoadjuvant treatment strategies for HER2-positive breast cancer: cost-effectiveness and quality of life outcomes. Breast Cancer Res Treat. (2020) 181:43–51. doi: 10.1007/s10549-020-05587-5, PMID: 32185586

[17] Gradishar WJ Moran MS Abraham J Abramson V Aft R Agnese D. Breast cancer, version 3.2024, NCCN clinical practice guidelines in oncology. J Natl Compr Canc Netw. (2024) 22:331–57. doi: 10.6004/jnccn.2024.0035, PMID: 39019058

[18] Ogston KN Miller ID Payne S Hutcheon AW Sarkar TK Smith I. A new histological grading system to assess response of breast cancers to primary chemotherapy: prognostic significance and survival. Breast. (2003) 12:320–7. doi: 10.1016/S0960-9776(03)00106-1, PMID: 14659147

[19] Choong GM Cullen GD O’Sullivan CC. Evolving standards of care and new challenges in the management of HER2-positive breast cancer. CA Cancer J Clin. (2020) 70:355–74. doi: 10.3322/caac.21634, PMID: 32813307

[20] Gerdes J Schwab U Lemke H Stein H. Production of a mouse monoclonal antibody reactive with a human nuclear antigen associated with cell proliferation. Int J Cancer. (1983) 31:13–20. doi: 10.1002/ijc.2910310104, PMID: 6339421

[21] Bae SJ Kim JH Lee MJ Baek SH Kook Y Ahn SG. Predictive markers of treatment response to neoadjuvant systemic therapy with dual HER2-blockade. Cancers. (2024) 16:842. doi: 10.3390/cancers16040842, PMID: 38398233 PMC10886516

[22] Lillemoe TJ Rendi M Tsai ML Knaack M Yarosh R Grimm E. HER2 testing characteristics can predict residual cancer burden following neoadjuvant chemotherapy in HER2-positive breast cancer. Int J Breast Cancer. (2021) 2021:6684629. doi: 10.1155/2021/6684629, PMID: 34123431 PMC8166502

[23] Liu T Lin J Lou J. Clinical application value of multi ⁃ parameter MRI radiomics evaluation of HER ⁃ 2 expression status in invasive breast cancer. J Nanjing Med University(Natural Sciences). (2024) 44:218–27. doi: 10.7655/NYDXBNSN230584

[24] Tanei T Seno S Sota Y Hatano T Kitahara Y Abe K. High HER2 intratumoral heterogeneity is a predictive factor for poor prognosis in early-stage and locally advanced HER2-positive breast cancer. Cancers. (2024) 16:1062. doi: 10.3390/cancers16051062, PMID: 38473420 PMC10930968

[25] Wang X Ba R Huang Y Cao Y Chen H Xu H. Time-dependent diffusion MRI helps predict molecular subtypes and treatment response to neoadjuvant chemotherapy in breast cancer. Radiology. (2024) 313:e240288. doi: 10.1148/radiol.240288, PMID: 39436292

[26] Ye G Wu G Zhang C Wang M Liu H Song E. CT-based quantification of intratumoral heterogeneity for predicting pathologic complete response to neoadjuvant immunochemotherapy in non-small cell lung cancer. Front Immunol. (2024) 15:1414954. doi: 10.3389/fimmu.2024.1414954, PMID: 38933281 PMC11199789

[27] Ren J Qi M Yuan Y Duan S Tao X. Machine learning-based MRI texture analysis to predict the histologic grade of oral squamous cell carcinoma. AJR Am J Roentgenol. (2020) 215:1184–90. doi: 10.2214/AJR.19.22593, PMID: 32930606

