# Supplementary material for: Developing a predictive model for neoadjuvant therapy in HER2 overexpression breast cancer using multi-parameter MRI radiomics: two-center retrospective study
Source: Front Oncol. 2025 Jul 15;15:1544058. doi: 10.3389/fonc.2025.1544058 (PMC12303799; doi:10.3389/fonc.2025.1544058)
Supplement: Supplementary file 1 [file Table1.docx]

**Supplementary Materials**

**Supplemental Material Table S1**

Center 1: Detailed MRI parameters of the First Affiliated Hospital of Bengbu Medical University

| Parameters | T1WI | T2WI | DWI | DCE |
| --- | --- | --- | --- | --- |
| Sequence | TSE | TSE | SE-EPI | 3D FFE |
| Fat suppression | None | SPAIR | SPAIR | SPAIR |
| Repetition time(ms) | 400 | 5000 | 2500 | 4.5 |
| Echo time(ms) | 10 | 60 | 42 | 2.2 |
| Flip angle(^0^) | 90 | 90 | 90 | 10 |
| Matrix | 124×131 | 236×279 | 176×167 | 228×301 |
| Field of view(mm) | 248×336 | 248×332 | 354×338 | 272×333 |
| Slice thickness(mm) | 4 | 4 | 4 | 4 |
| b-values(sec/mm^2^) | None | None | 0/800 | None |

**Supplemental Material Table S2**

Center 2: Detailed MRI parameters of Daping Hospital, Army Military Medical University

| Parameters | T1WI | T2WI | DWI | DCE |
| --- | --- | --- | --- | --- |
| Sequence | SE | FSE | SE-EPI | FFE |
| Fat suppression | None | FS | FS | FS |
| Repetition time(ms) | 8.6 | 5600 | 3300 | 4.62 |
| Echo time(ms) | 4.7 | 57 | 94 | 1.75 |
| Flip angle(^0^) | 90 | 90 | 90 | 10 |
| Matrix | 126×132 | 230×270 | 128×128 | 384×320 |
| Field of view(mm) | 320×320 | 320×320 | 320×320 | 360×360 |
| Slice thickness(mm) | 5 | 5 | 5 | 1.5 |
| b-values(sec/mm^2^) | None | None | 0/1000 | None |
|  |  |  |  |  |
